# The OPS-SAT benchmark for detecting anomalies in satellite telemetry

**DOI:** 10.1038/s41597-025-05035-3

**Published:** 2025-04-29

**Authors:** Bogdan Ruszczak, Krzysztof Kotowski, David Evans, Jakub Nalepa

**Affiliations:** 1 Computer Science Department, Opole University of Technology, Prószkowska Str. 76, 45-758 Opole, Poland; 2 KP Labs, Bojkowska Str. 37J, 44-100 Gliwice, Poland; 3 European Space Agency/ESOC, Robert-Bosch-Str. 5, 64293 Darmstadt, Germany; 4 Faculty of Automatic Control, Electronics and Computer Science, Department of Algorithmics and Software, Silesian University of Technology, Akademicka Str. 16, 44-100 Gliwice, Poland

**Keywords:** Computational science, Information technology

## Abstract

Detecting anomalous events in satellite telemetry is a critical task in space operations. It is time-consuming, error-prone and human dependent, thus automated data-driven algorithms have been emerging at a steady pace. However, there are no available datasets of real satellite telemetry with annotations to verify anomaly detection models. We address this gap and introduce the AI-ready benchmark dataset (OPSSAT-AD) containing the telemetries acquired on board OPS-SAT—a CubeSat mission, operated by the European Space Agency. The dataset is accompanied with the baseline results obtained using 30 supervised and unsupervised classic and deep machine learning algorithms. They were evaluated using the training-test dataset split introduced in this work, and we suggest a set of quality metrics which should be calculated to confront the new algorithms for anomaly detection while exploiting OPSSAT-AD. We believe that this work may become an important step toward building a fair, reproducible and objective validation procedure that can be used to quantify the capabilities of the emerging techniques in an unbiased and fully transparent way.

## Background & Summary

The anomaly detection (AD) domain encompasses a diverse array of methodologies for the identification of anomalous patterns in data of various modalities. These approaches can be applied to a multitude of data types, including images, text, and time series data, among others. However, the development and evaluation of real-world anomaly detection applications are dependent on the availability of real-world data. Currently, there is a considerable number of datasets available for a wide range of scenarios^1^, but the satellite telemetry data for AD is an extremely underrepresented category in this catalogue. This kind of data is difficult and costly to obtain, often confidential, and requires expert knowledge to annotate properly. The only two widely accessible and used collections of this type include the NASA Soil Moisture Active Passive (SMAP) and Mars Science Laboratory (MSL) datasets^2^. They offer short fragments of signals and related commands from 55 and 27 telemetry parameters, respectively, with a total of 105 annotated anomalies. However, the recent consensus in the community is that they should not be used for time series AD benchmarking due to their unrealistic anomaly density, many trivial anomalies, mislabelled ground truth, distributional shifts, and a lack of meaningful correlation between commands and channels^3–5^. Other well-known satellite telemetry datasets, such as Mars Express^6^ or NASA WebTCAD^7^, do not contain annotations of anomalous events. There is an ongoing activity to publish a large-scale AD dataset by European Space Agency (ESA) solving all the mentioned issues^8,9^, but it will primarily address the needs of large-scale, complex and relatively stable missions.

The dataset introduced in this article, dubbed OPSSAT-AD, is fundamentally different from those available in the literature, as it tackles a very specific ESA OPS-SAT mission—a CubeSat flying laboratory, for which we might expect a noticeable number of abnormal events^10^. The raw telemetry from OPS-SAT is characterized by many data gaps, artifacts, sampling frequency changes, and signal amplitude variations. The dataset was collectively curated by space operations engineers and machine learning experts to make it useful for building and validating data-driven anomaly detection techniques. It includes a selection and the corresponding ground-truth annotation of 2123 short single-channel satellite telemetry fragments (univariate time series) captured within 9 telemetry channels. Due to the underlying nature of the OPS-SAT mission, anomalous fragments account for 20% of the dataset. Such fragments contain raw data with many aforementioned real-life challenges, and they differ in their length and sampling frequency. For each telemetry fragment, the dataset also contains a set of 18 handcrafted features used in the actual machine learning AD algorithm validated on board OPS-SAT^11^. These features are exploited in this article to benchmark 30 other supervised and unsupervised machine learning algorithms for anomaly detection. All of them were trained on 1494 and tested on 529 telemetry segments, and assessed using 7 metrics suggested for quantifying the operational capabilities of anomaly detection algorithms—this training-test dataset split is included in our benchmark as well.

Overall, the benchmark (including the dataset, training-test dataset split, suggested quality metrics, and our baseline results) introduced in this paper shall help the community to create and compare their approaches to detecting anomalies in real-life satellite telemetry in a fair and unbiased way. Therefore, we also address the reproducibility crisis currently observed in the (not only) machine learning community^12^. While the OPS-SAT spacecraft completed its atmospheric reentry at the end of May 2024, its successor—OPS-SAT VOLT—is going to be launched in late 2025 and will make a great opportunity to validate the algorithms developed based on our benchmark in the wild after deploying them on-board an operational satellite. Although future satellites may be equipped with different sensor sets, with possible variations in sampling rates or data ranges, and other telemetry may be important to monitor, we believe that a collection of real data such as the one examined in this paper is of great value to the community interested in on-board deployments.

## Methods

### Data acquisition and annotation

**Fig. 1:**
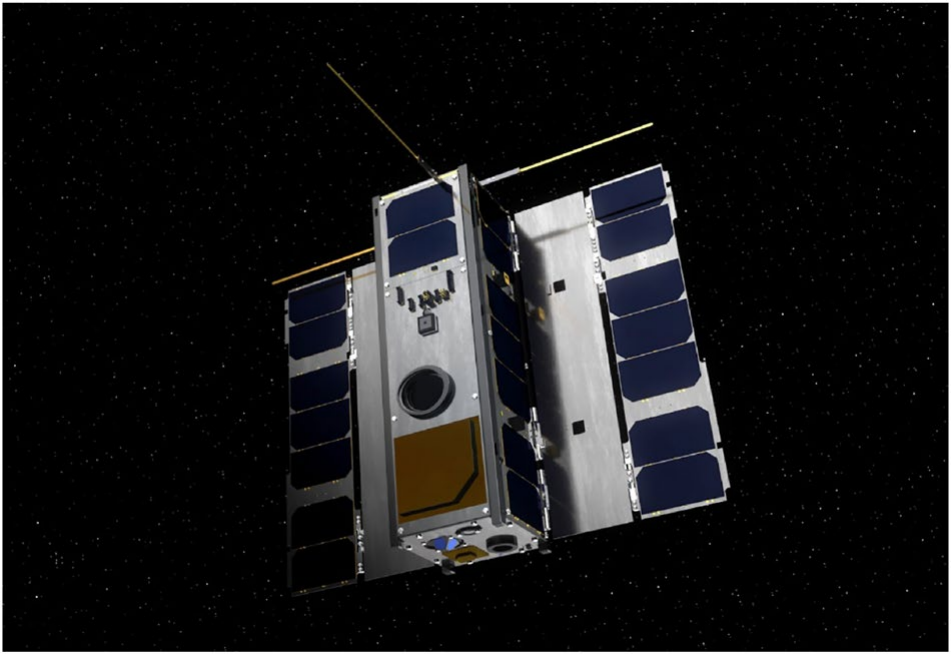
The ESA OPS-SAT frontal view. Image credits: European Space Agency.

The telemetry data delivered^13^ in this paper was acquired from the ESA OPS-SAT satellite (Fig. 1). It is a small 3-unit (3U, where 1U=10 cm^3^) CubeSat launched in December 2019 with the primary objective of being a technological demonstrator for in-orbit data processing. It finished its mission with the atmospheric reentry on 22 May 2024, but it generated lots of useful data during more than 4 years of its operations, including satellite imagery^14^ and telemetry^11^.

OPS-SAT offered a unique opportunity for researchers to run their experiments and algorithms in orbit. While these experiments were carried out, all telemetry data was simultaneously collected and recorded in the ESA archive. The archive was monitored for potential anomalies to ensure the mission’s stable and uninterrupted operation. Our dataset consists of telemetry fragments recommended by the OPS-SAT operation engineers as the most “interesting” (according to their subjective assessment) for anomaly detection. The actual data collection process was carried out using the data exchange platform WebMUST^15^ used in the European Space Operations Centre (ESOC). This platform is restricted to the authorized ESA partners only, but the data included in our dataset package does not have to be requested through it, and thus is made publicly available.

The online OXI tool for visualization and annotation of satellite telemetry (https://oxi.kplabs.pl/)^16^ was used to enable a collaborative labeling process of the dataset. Using this application, domain experts were able to manually extract and annotate telemetry segments representing periods of nominal and anomalous operation. The initial selection of anomalies was provided by 3 ESA spacecraft operations engineers and further curated by 2 machine learning experts (with more than 10 years of experience each). The curated annotations were finally reviewed by the three spacecraft operations engineers. The detailed satellite telemetry annotation process, together with the visual artefacts generated throughout it, are discussed in^11,17,18^.

The telemetry segments have been designated as anomalous for the cases that the operators should be notified about. The anomalous segments included in the collection are characterized by instances of unusual signal shapes, unexpected sensor behavior (e.g., single or multiple peaks), signal fragments with zero values, or gaps in the signal. In such cases, it is imperative to initiate the shutdown or restart of pertinent aircraft systems or sensors, or alternatively, to transition their operation to safe mode.

### Feature extraction

Due to the characteristics of satellite telemetry, the segments of raw data selected by the domain experts have varying lengths and sampling frequency. As such, they could not be handled by most machine learning algorithms without performing an additional preprocessing or feature extraction. Thus, 18 handcrafted features were designed for the task of anomaly detection^11^—they were calculated separately for each segment, and they are included in our benchmark. An algorithm operating on such features was already validated in our previous work focusing on the application of data-driven anomaly detection on board OPS-SAT^11^.

**Fig. 2:**
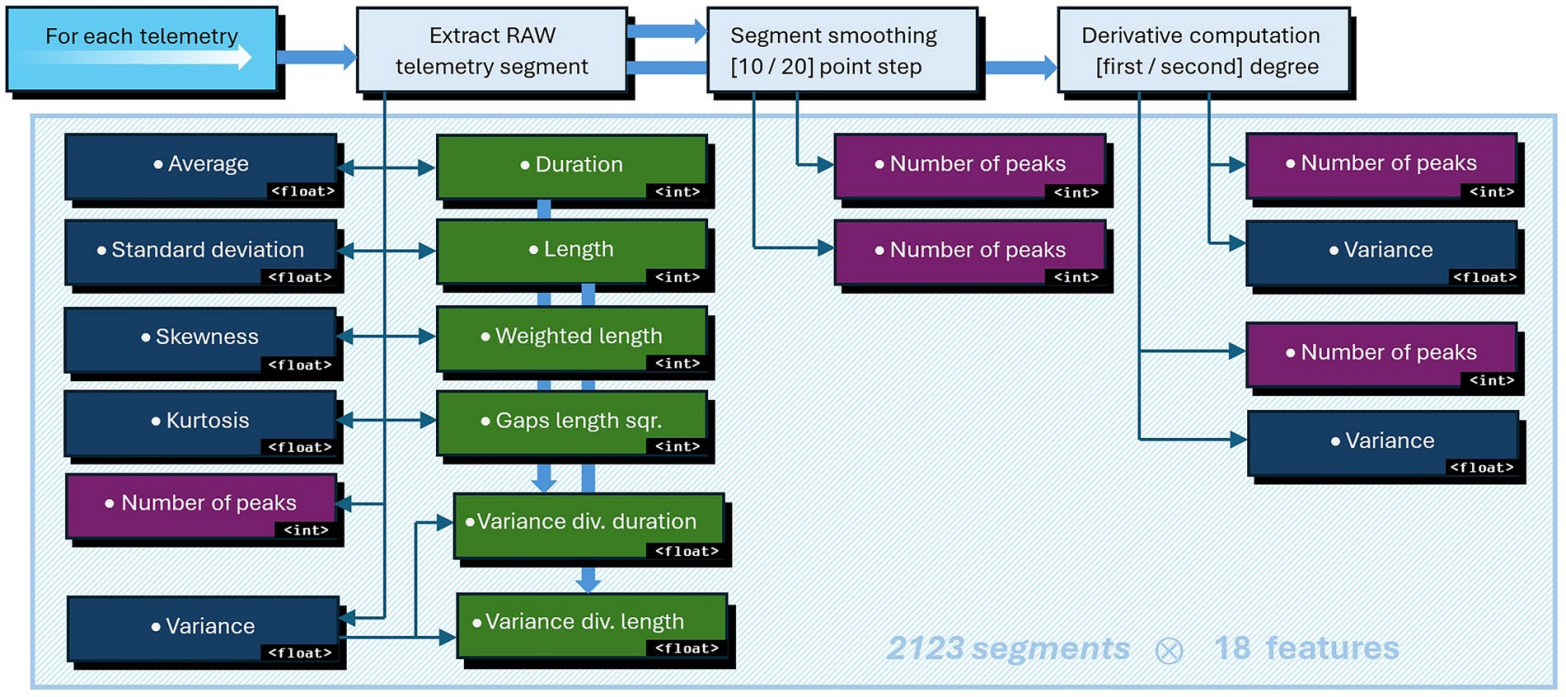
The features extracted for each segment, with the corresponding data type. The meaning of the colors: dark blue for popular statistics, violet for peak counters for various converted segments, and green for the length or duration-related features.

The features extracted for each telemetry segment are presented in Fig. 2. They are divided into three groups:

- **12 features extracted from raw segments**, including basic statistics, such as the arithmetic average of the signal values, their standard deviation, skewness, kurtosis and variance (⟨*mean*⟩, ⟨*std*⟩, ⟨*skew*⟩, ⟨*kurtosis*⟩, and ⟨*var*⟩), but also the number of peaks (of the minimum of 10% prominence, with a peak prominence measuring how much a peak “stands out” in relation to the signal, while considering its height and location: ⟨*n*_*peaks*⟩), duration (in seconds: ⟨*duration*⟩) and the length (in the number of telemetry points: ⟨*len*⟩), the weighted length (weighted by sampling: ⟨*len*_*weighted*⟩), the gaps’ length (the squared number of missing data points: ⟨*gaps*_*squared*⟩), and the weighted variance (weighted by the duration and by the length: ⟨*var*_*div*_*duration*⟩, ⟨*var*_*div*_*len*⟩).
- **2 features extracted from the smoothed segments** (**using the uniform interpolation**^19^), including the number of peaks (extracted using the 10 and 20 points smoothing steps: ⟨*smooth*10_*n*_*peaks*⟩, ⟨*smooth*20_*n*_*peak*⟩).
- **4 features extracted from the first and the second derivatives of the segment**, including the number of peaks and variance (⟨*diff*_*peaks*⟩, ⟨*diff*2_*peaks*⟩, ⟨*diff*_*var*⟩, ⟨*diff*2_*var*⟩).

Employing the duration, the length and the gaps’ length features should allow the algorithms to easily capture some “obvious” abnormalities in the telemetry data. This intuitively could lead to promote some less computationally demanding AD methods suitable for on-board applications. The proposed set of features serves as an example and may be easily expanded (or replaced) by the community by (*i*) designing new feature extractors (potentially followed by feature selectors), (*ii*) using other well-established feature sets^20^ or (*iii*) benefiting from the automated feature learning^21^.

**Fig. 3:**
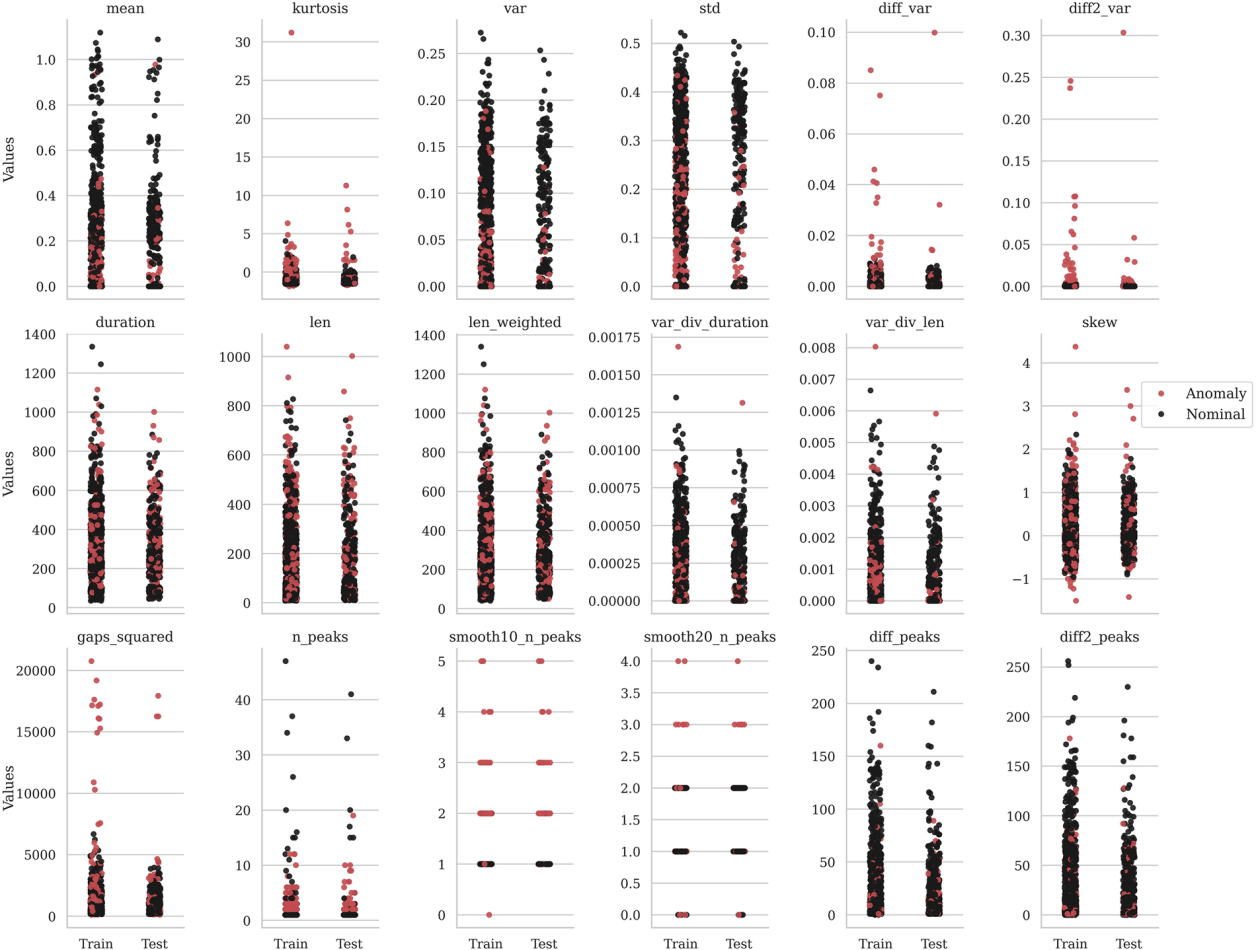
Dataset features for the training and validation subsets with the indication of anomalies (marked in red).

**Fig. 4:**
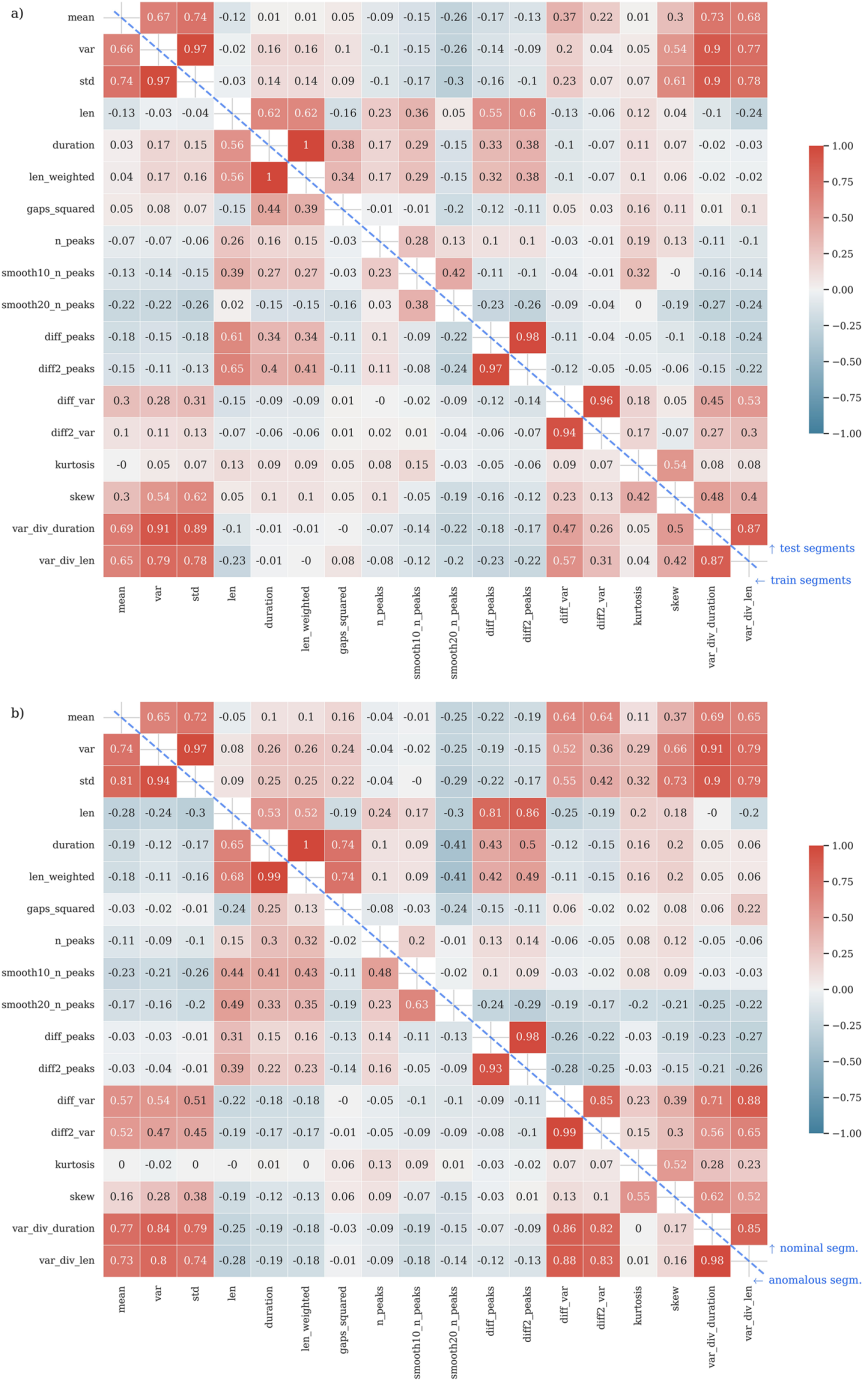
The coefficient of correlation computed between each feature, for the training (below the blue dashed diagonal line) and test set (above the same diagonal line). We employed: (**a**) Pearson’s correlation coefficient and (**b**) Spearman’s Rank correlation coefficient.

The distribution of the extracted features elaborated for each telemetry segment included in our dataset is depicted in Fig. 3. We compare the distribution for the training (${\boldsymbol{T}}$) and test (Ψ) sets, to visualize the effect of the training-test dataset split on the feature distributions. Figure 4 provides a detailed view of the relations between the extracted features for the ${\boldsymbol{T}}$ and Ψ sets. We rendered this plot to confirm that both subsets represent similar data distributions.

### Benchmarking procedure

We provide a procedure that should be followed to confront the AD algorithms over our dataset. The entire dataset of 2123 telemetry segments is split into the training (${\boldsymbol{T}}$) and test (Ψ) sets (Table 1), forming an AI-ready dataset. To extract these subsets, we performed stratified random sampling to maintain the original percentage of anomalies in both ${\boldsymbol{T}}$ and Ψ.

**Table 1:** The number of telemetry segments included in the training (${\boldsymbol{T}}$) and test (Ψ) sets.

Class	Training set (${\boldsymbol{T}}$)	Test set (Ψ)	Total
Nominal	1273	416	1689
Anomalous	321	113	434
Sum	1494	529	2123

The benchmarking procedure can be summarized by the following steps:

1. Load the dataset from the dataset.csv file.
2. Split the dataset into ${\boldsymbol{T}}$ and Ψ according to the ⟨*train*⟩ attribute included in this file.
3. **[Optionally]** Preprocess the datasets using e.g., data normalization, additional feature extraction, feature selection and other steps directly related to the AD algorithm which undergoes the benchmarking process.
4. **[Optionally]** Train a machine learning model over ${\boldsymbol{T}}$.
5. Quantify the algorithm’s performance over Ψ using the metrics discussed in the next section.

### Quality metrics

The following metrics should always be calculated over the test set Ψ while confronting the AD algorithms (both supervised and unsupervised) over the dataset OPSSAT-AD introduced in this work:

- **Accuracy:** (*TP* + *TN*)/(*TP* + *TN* + *FP* + *FN*),
- **Precision:*** TP*/(*TP* + *FP*),
- **Recall:***TP*/(*TP* + *FN*),
- **F**_1_ **score**: (2 ⋅ precision ⋅ recall)/(precision + recall),
- **Matthews’ Correlation Coefficient (MCC)**^22^: $$(TP\cdot TN-FP\cdot FN)/$$$$\sqrt{(TP+FP)\cdot (TP+FN)\cdot (TN+FP)\cdot (TN+FN)}$$,
- **Area under the receiver operating characteristic curve** (**AUC**_**ROC**_),
- **Area under the precision-recall curve** (**AUC**_**PR**_),

Where *TP*, *TN*, *FP*, and *FN* are the number of true positives (anomalous telemetry segments correctly identified as anomalies), true negatives (nominal telemetry segments correctly identified as nominal), false positives (nominal telemetry segments incorrectly identified as anomalies), and false negatives (anomalous telemetry segments incorrectly identified as nominal). All metrics should be maximized (*↑*), with one indicating the best score (MCC ranges from  − 1 to 1, other metrics from 0 to 1).

### The baseline: anomaly detection algorithms

Although there are ground-truth AD datasets that may be used to train supervised models for this task, they are extremely limited and, by definition, they cannot capture a representative set of anomalies (otherwise such “anomalies” would not be “anomalies” any longer). In practice, while building data-driven AD algorithms for satellite telemetry, practitioners may not be able to access real-life ground-truth data, hence unsupervised methods have been gaining research attention. Here, we establish a set of baseline results obtained using 30 AD methods, including both supervised and unsupervised algorithms (Table 2).

**Table 2:** Anomaly detection methods investigated in this study.

Abbreviation	Year	Algorithm
***Supervised algorithms***		
Linear+L2^30^	2006	Linear classifier with *L*_2_ regularization
LR^31^	2008	Logistic regression
AdaBoost^32^	2009	Adaptive Boosting
LSVC^33^	2013	Support Vector Classifier with the squared hinge linear loss
XGBOD^34^	2018	Extreme Gradient Boosting Outlier Detection
FCNN^35^	2019	Fully Connected Neural Network with dropout and batch normalization
RF+ICCS^11^	2023	Random Forest based model with segment augmentation
***Unsupervised algorithms***		
PCA^36,37^	1996	Principal Component Analysis
LMDD^38^	1996	Linear Method for Deviation Detection
COF^39^	2002	Connectivity-based Outlier Factor
KNN^40^	2002	K-Nearest Neighbors
CBLOF^41^	2003	Cluster-Based Local Outlier Factor
ABOD^42^	2008	Angle-based Outlier Detector
IForest^43^	2008	Isolation Forest
SOD^44^	2009	Outlier Detection in Axis-Parallel Subspaces of High Dimensional Data
SOS^45^	2012	Stochastic Outlier Selection
VAE^46^	2013	Variational Autoencoder
OCSVM^47^	2016	One-Class Support Vector Machine with a polynomial kernel
LODA^48^	2016	Lightweight On-line Detector of Anomalies
GMM^37^	2017	Gaussian Mixture Model
AnoGAN^49^	2017	Generative Adversarial Networks for AD
DeepSVDD^50^	2018	Deep one-class classification
ALAD^51^	2018	Generative Adversarial Networks for AD
INNE^52^	2018	Isolation-based Anomaly Detection Using Nearest-Neighbor Ensembles
SO-GAAL^53^	2020	Single-objective Generative Adversarial Active Learning
MO-GAAL^53^	2020	Multi-objective Generative Adversarial Active Learning
COPOD^54^	2020	Copula-based outlier detection
ECOD^55^	2022	Empirical Cumulative Distribution Functions
LUNAR^56^	2022	Unified Local Outlier Detection with Graph Neural Networks
DIF^57^	2023	Deep Isolation Forest

For all those algorithms that we implemented in the PyOD framework^23^ (https://pyod.readthedocs.io/en/latest/), the default parameters (suggested by the authors of these techniques) are used, with the anomaly contamination factor set to 0.2, according to the anomaly distribution observed in ${\boldsymbol{T}}$. To ensure reproducibility, we provide a Jupyter Notebook showing how to execute an example AD algorithm, in a both supervised and unsupervised training regime (*modeling_examples.ipynb*). We note that for the supervised algorithms we employed (denoted in Table 2 as AdaBoost, FCNN, Linear+L2, LR, LSVC, XGBOD, RF+ICCS) the contamination parameter is not exploited.

## Data Records

**Fig. 5:**
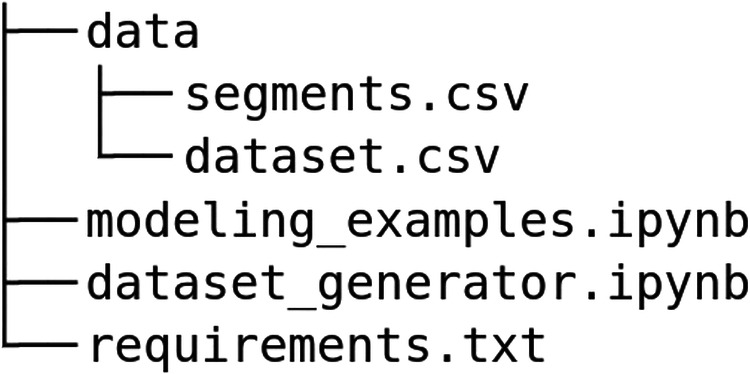
The layout of the OPS-SAT benchmark for anomaly detection.

We published the OPSSAT-AD benchmark dataset at Zenodo^13^. It is built of 9 source telemetry channels that were selected by the space operations engineers. They include 3 magnetometer telemetry channels: I_B_FB_MM_0 (CADC0872), I_B_FB_MM_1 (CADC0873), I_B_FB_MM_2 (CADC0874), and 6 photo diode (PD) channels: I_PD1_THETA (CADC0884), I_PD2_THETA (CADC0886), I_PD3_THETA (CADC0888), I_PD4_THETA (CADC0890), I_PD5_THETA (CADC0892), I_PD6_THETA (CADC0894). Here, the names correspond to the source names from the WebMUST repository and the OPS-SAT telemetry channel names (in brackets). The layout of the dataset is summarized in Fig. 5—it includes both the raw files, as well as the extracted features in a tabular form.

### Raw telemetry data

**Fig. 6:**
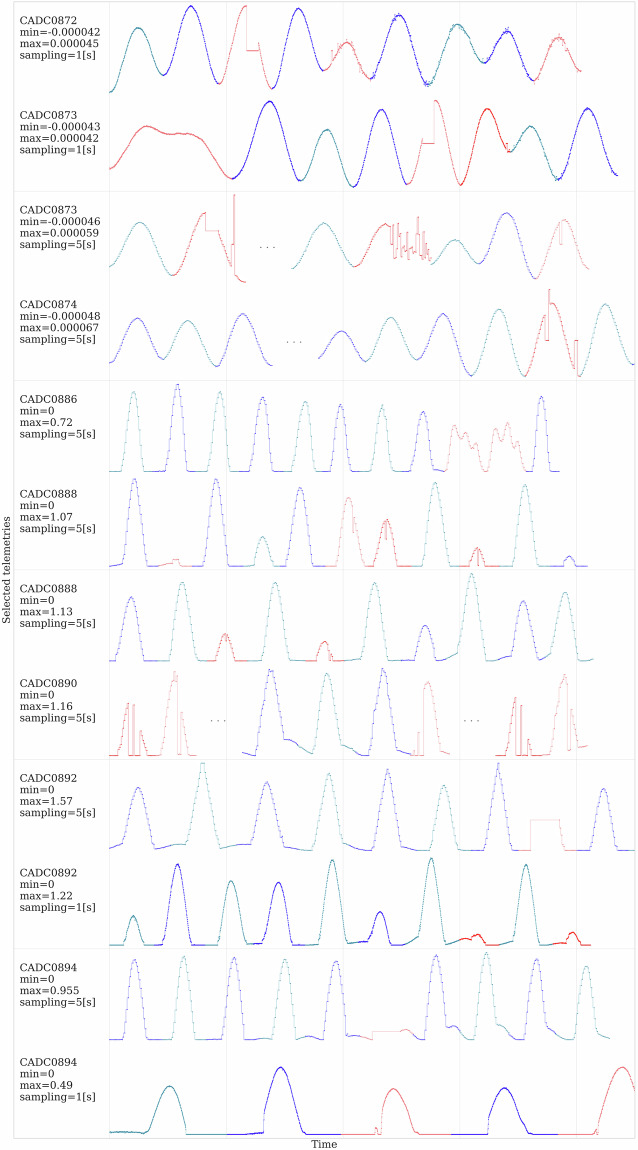
Selected segments from out OPS-SAT dataset. Several types of signal distortions are depicted, including peaks, deformations, noise (CADC0873), irregular periodicity (CADC0886), short (CADC0892, CADC0894) and long data gaps (CADC0874). Anomalous segments are plotted in red. For brevity, we omit the axis values, but provide data ranges and the sampling information for each channel.

In Fig. 6, we visualize selected characteristics of the acquired telemetry signals, effectively showing real-world challenges concerned with telemetry data acquired in the wild (e.g., missing readouts, different sampling frequencies). Such segments for all the aforementioned telemetry channels and their selected parts are included in the data/segments.csv file. It contains the attributes that identify the registration time: ⟨*timestamp*⟩ (ISO date format), ⟨*channel*⟩ (the channel name), ⟨*value*⟩ (the acquired signal value), and ⟨*label*⟩ (the ground-truth annotation). Additionally, we provide the consecutive segment numbers (⟨*segment*⟩), their sampling rate (⟨*sampling*⟩), and the indication if they are included in ${\boldsymbol{T}}$ (⟨*train*⟩).

### Extracted features

In the tabular version of the dataset (data/dataset.csv), we include the extracted features. In the Supplementary Materials, we present the distributions of all of the provided features, rendered for both ${\boldsymbol{T}}$ and Ψ sets of our dataset (Fig. 3).

## Technical Validation

### Universal anomaly detectors

For this paper, we perform two alternative series of verifications that resemble two alternative approaches toward building anomaly detectors. We first evaluate a battery of machine learning models in a universal detector scenario, in which an anomaly detection algorithm should allow for monitoring a number of different telemetry channels using the proposed set of intermediate features. Secondly, we implemented and evaluated a set of specialized (channel-wise) detectors, for which we first prepared a separate model for each telemetry channel and aggregated their responses later.

**Fig. 7:**
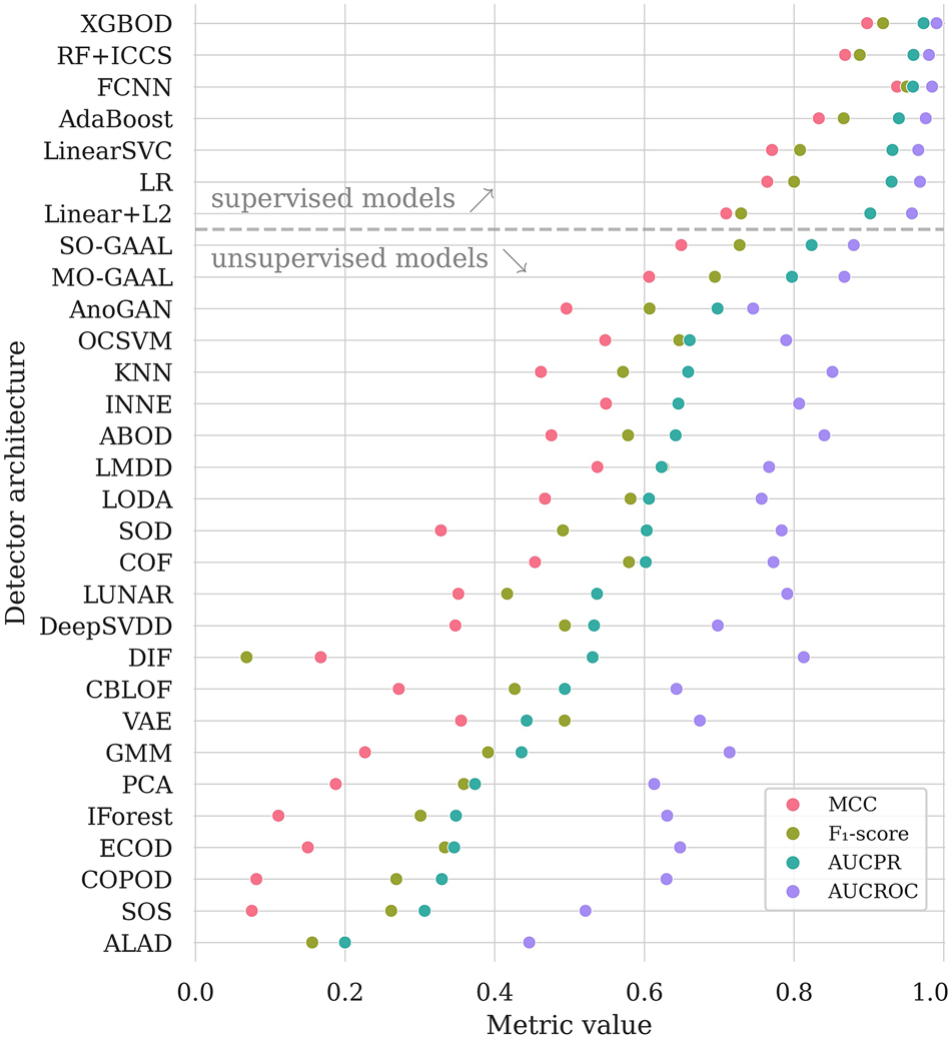
The results (over Ψ) obtained using the investigated machine learning models (first grouped according to their training strategy, either supervised or unsupervised, then sorted by *F*_1_).

**Fig. 8:**
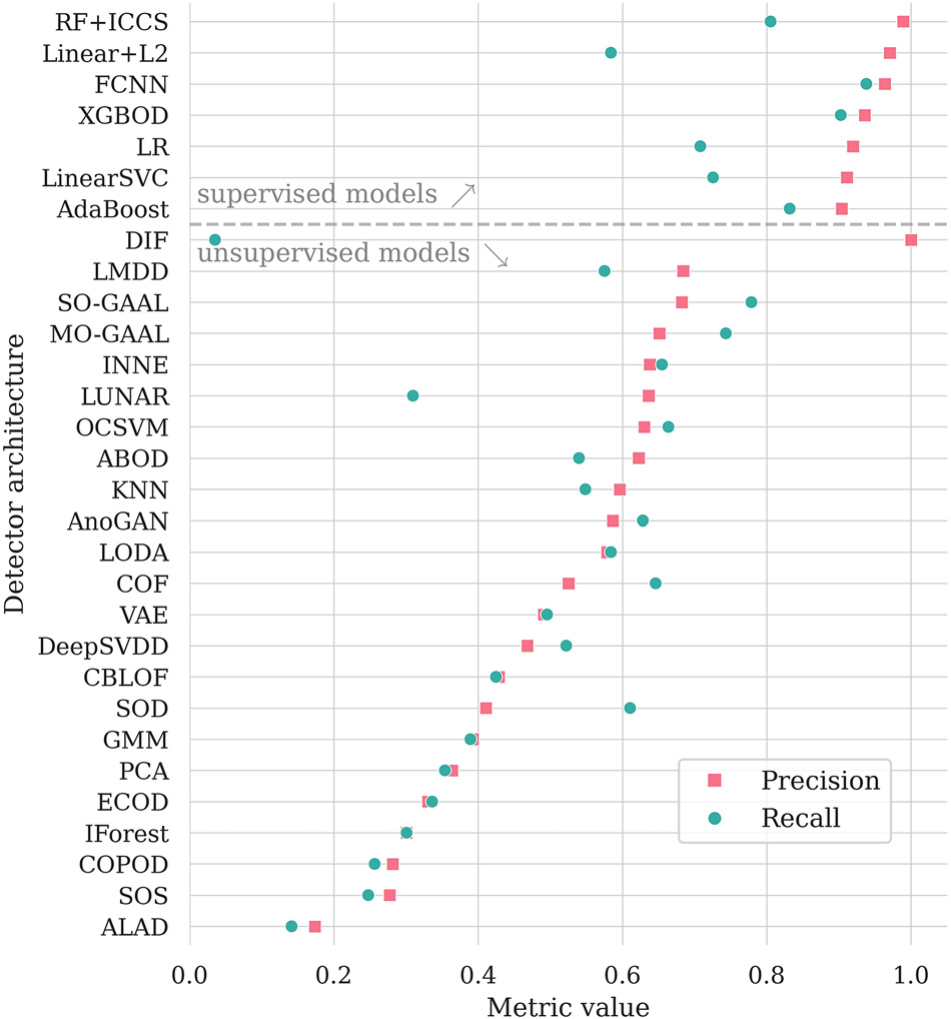
Precision and recall metrics (over Ψ) obtained using the investigated algorithms. Models are sorted according to the number of misclassified telemetry segments, with the best-performing one rendered on top of the graph.

In Table 3, we aggregate the results obtained using all the investigated AD algorithms. Here, we highlighted the globally best results (in bold for each quality metric), and we underlined the best results elaborated by the unsupervised algorithms, as we consider them a different category of the AD solutions. We are aware that some algorithms from PyOD should be rather trained using nominal data only (i.e., OCSVM or autoencoders) to achieve better results. As an example, the OCSVM model achieves *AUC*_*PR*_ of 0.659 and *AUC*_*ROC*_ of 0.787 in our setting, but when using only the nominal data (without abnormal segments) for training, the corresponding values are 0.762 and 0.815. However, we wanted our baseline to be consistent and to reflect a typical usage of the PyOD framework by a non-expert user. Also, the fine-tuning of those algorithms is out of the scope of this study. In Fig. 7, we render the selected metrics for each model. We can indeed observe the better performance of supervised methods, as those could actively benefit from the labeled anomaly examples while building a machine learning model. In Fig. 8, we also display the precision and recall quality metrics. For the fully-connected neural network, we can observe only four false positives and eight false negatives of all Ψ samples, reaching the precision of 0.963, and the recall of 0.929.

**Table 3:** The experimental results for the universal detector, sorted by *AUC*_*PR*_.

Model	*AUC*_*PR*_ (↑)	*AUC*_*ROC*_ (↑)	*Accuracy* (↑)	*F*_1_ (↑)	*Precision* (↑)	*Recall* (↑)	*MCC* (↑)
***Supervised algorithms***							
FCNN	**0.979**	0.989	**0.977**	**0.946**	0.963	**0.929**	**0.932**
XGBOD	0.975	**0.992**	0.966	0.918	0.944	0.894	0.897
RF+ICCS	0.963	0.985	0.955	0.883	0.978	0.805	0.862
LSVC	0.934	0.968	0.926	0.808	0.911	0.726	0.771
LR	0.931	0.969	0.924	0.800	0.920	0.708	0.764
AdaBoost	0.923	0.962	0.934	0.836	0.890	0.788	0.797
Linear+L2	0.901	0.958	0.905	0.722	0.970	0.575	0.703
***Unsupervised algorithms***							
MO-GAAL	0.779	0.865	0.907	0.726	0.985	0.575	0.710
AnoGAN	0.668	0.756	0.868	0.588	0.877	0.442	0.563
SO-GAAL	0.660	0.749	0.885	0.655	0.906	0.513	0.627
OCSVM	0.659	0.787	0.845	0.647	0.630	0.664	0.548
KNN	0.658	0.852	0.824	0.575	0.594	0.558	0.465
ABOD	0.644	0.843	0.832	0.582	0.620	0.549	0.479
INNE	0.643	0.806	0.847	0.646	0.638	0.655	0.549
ALAD	0.629	0.744	0.870	0.596	0.879	0.451	0.570
LMDD	0.623	0.767	0.854	0.628	0.691	0.575	0.542
SOD	0.621	0.797	0.737	0.505	0.423	0.628	0.348
COF	0.603	0.774	0.794	0.576	0.514	0.655	0.448
LODA	0.597	0.748	0.822	0.588	0.583	0.593	0.475
LUNAR	0.540	0.792	0.813	0.407	0.630	0.301	0.342
CBLOF	0.493	0.642	0.756	0.427	0.429	0.425	0.272
DIF	0.465	0.797	0.790	0.035	$\underline{{\bf{1.000}}}$	0.018	0.118
VAE	0.450	0.680	0.796	0.349	0.547	0.257	0.272
GMM	0.426	0.713	0.737	0.393	0.388	0.398	0.225
DeepSVDD	0.375	0.610	0.775	0.279	0.442	0.204	0.184
PCA	0.373	0.612	0.728	0.357	0.360	0.354	0.185
IForest	0.347	0.635	0.701	0.295	0.297	0.292	0.105
ECOD	0.340	0.637	0.720	0.345	0.345	0.345	0.167
COPOD	0.328	0.627	0.703	0.270	0.284	0.257	0.084
SOS	0.308	0.524	0.705	0.264	0.283	0.248	0.081

The globally best results for each metric are **boldfaced**, and the best among the unsupervised algorithms are underlined.

The investigation of the unsupervised algorithms reveals that some of them reach a point where they return a small set of mistakenly assessed telemetry samples. Especially the detectors built upon MO-GAAL, SO-GAAL and AnoGAN offered high precision. In terms of the number of misclassified examples, MO-GAAL obtained a better result than the supervised methods, and made one less false detection when compared to FCNN. A number of other unsupervised algorithms, however, tend to either return a large number of false negatives, or to raise many false alarms with a low number of false negatives. In the first group of such methods, we can observe DIF, ALAD, DeepSVDD, and AnoGAN, whereas e.g., COF belongs to the second group here. The usability of such algorithms would be rather limited to situations when avoiding one type of the classification error could be more practically important (e.g., to minimize the overhead induced on the space operations teams that would have to review many incorrectly raised false alarms).

### Specialized anomaly detectors

To better reflect a real-life approach of building separate anomaly detectors for each telemetry channel, we follow this scenario in the second experiment. In this experiment, we utilize the same list of machine learning models, but we employed telemetry data from a narrowed list of OPS-SAT channels: CADC0872, CADC0873, CADC0874, CADC0888, CADC0892, and CADC0894. The other channels were filtered out, as they had no anomalous segments in the training or test subsets. Also, we use the same input data (*segments.csv*), but—as we perform the verification for each telemetry channel independently—the dataset which is exploited for each training and test round is a subset of the dataset used in the previous experiment. For the training sets, the number of nominal segments ranges from 95 (CADC0894) to 366 (CADC0873), and for anomalous segments from 16 (CADC0894) to 99 (CADC0872). We utilized the same data split to get the test subset, as in the previous experiments. The proportion of nominal and anomalous examples is similar, and specifically, the number of nominal segments in the test set ranges from 28 (CADC0894) to 122 (CADC0873), and for anomalous segments from 5 (CADC0894) to 32 (CADC0872).

Additionally, in this experiment, we do not utilize two features, i.e., len and duration, to make the analysis independent of the telemetry segment size. Also, we encourage the community to build new feature extractors. For this reason, the code we employed to convert the telemetry segments into a final dataset is included in the package delivered with this paper. The transformation of segments.csv could be reproduced, or modified, using the Jupyter notebook: dataset_generator.ipynb.

**Fig. 9:**
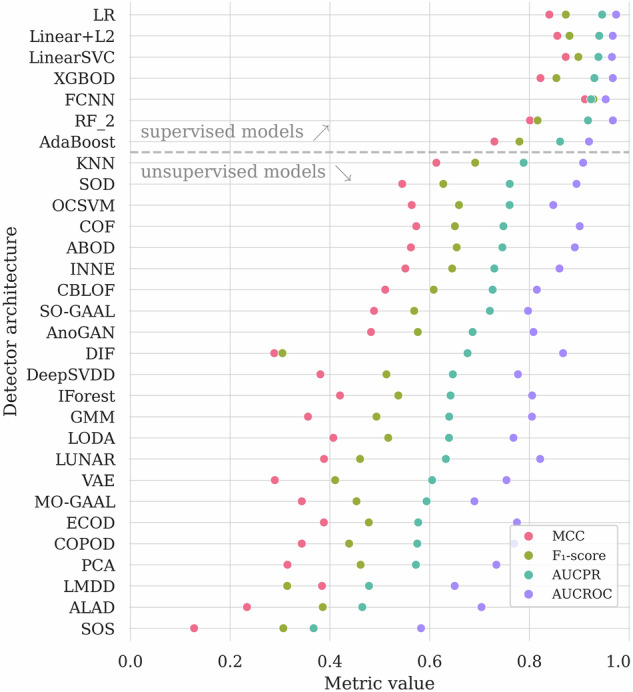
The results (over Ψ) obtained using the investigated machine learning models (first grouped according to their training strategy, either supervised or unsupervised, then sorted by *F*_1_).

**Fig. 10:**
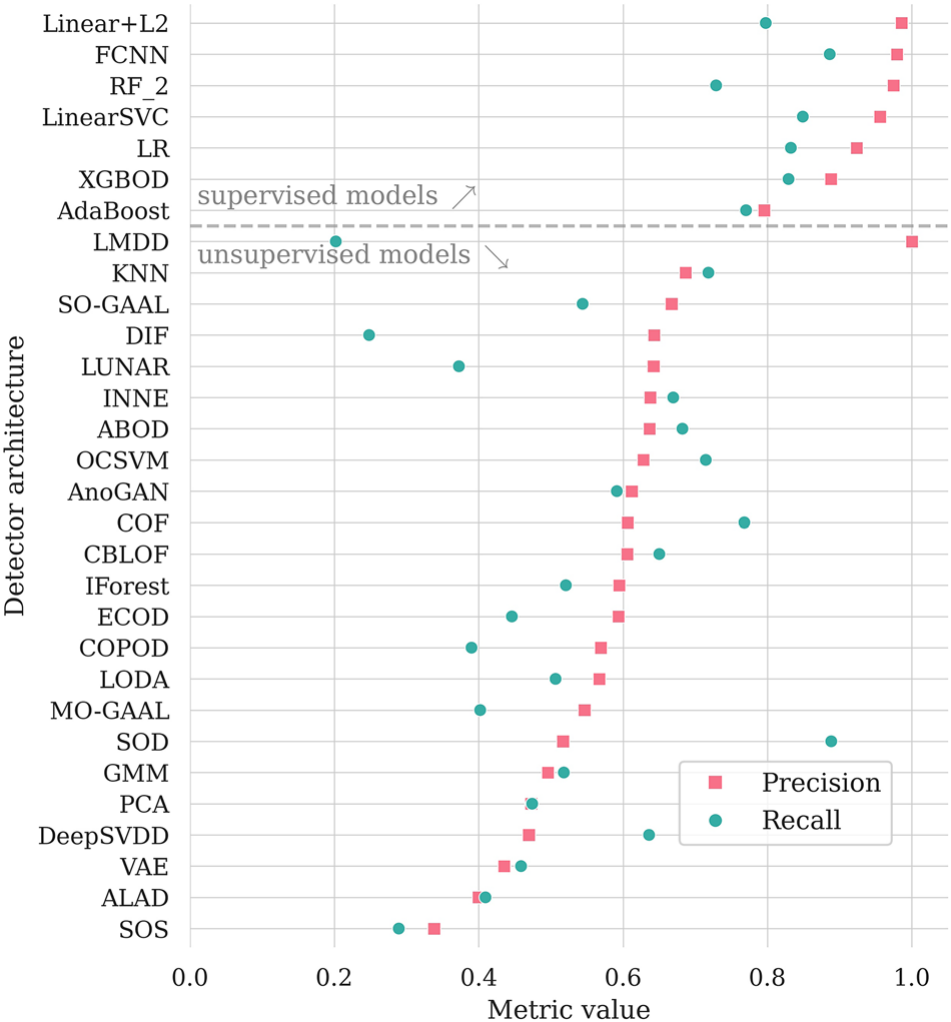
Precision and recall metrics (over Ψ) obtained using the investigated algorithms. Models are sorted according to the number of misclassified telemetry segments, with the best-performing one rendered on top of the graph.

The results for the specialized models are on a similar performance level as those obtained for the universal models, although the ranking of the solutions is different. For this evaluation, we get the largest *AUC*_*PR*_ and *AUC*_*ROC*_ for the linear regression-based models (0.946 and 0.974, respectively). However, the best detection accuracy, *F*_1_ score, and MCC of 0.970, 0.929, and 0.912, respectively, we noted for the fully-connected neural network, as in the previous experiment. For the unsupervised models, we found the KNN-based models with the greatest scores. We provide a detailed set of results in with the full results’ table (Table 4) and the figures depicting the metrics (Figs. 9 and 10).

**Table 4:** The experimental results for the specialized models, trained separately for each telemetry channel.

Model	*AUC*_*PR*_ (↑)	*AUC*_*ROC*_ (↑)	*Accuracy* (↑)	*F*_1_ (↑)	*Precision* (↑)	*Recall* (↑)	*MCC* (↑)
***Supervised algorithms***							
LR	**0.946**	**0.974**	0.944	0.873	0.924	0.832	0.840
Linear+L2	0.940	0.967	0.949	0.880	0.986	0.798	0.856
LinearSVC	0.939	0.966	0.957	0.898	0.956	0.849	0.873
XGBOD	0.930	0.967	0.945	0.854	0.888	0.829	0.822
FCNN	0.924	0.953	**0.970**	**0.929**	**0.979**	**0.886**	**0.912**
RF	0.918	0.967	0.939	0.816	0.975	0.729	0.801
AdaBoost	0.862	0.920	0.917	0.780	0.796	0.770	0.730
***Unsupervised algorithms***							
KNN	0.788	0.908	0.868	0.691	0.687	0.718	0.613
SOD	0.761	0.895	0.777	0.627	0.517	0.888	0.545
OCSVM	0.760	0.848	0.844	0.659	0.628	0.715	0.564
COF	0.748	0.901	0.832	0.651	0.606	0.768	0.573
ABOD	0.746	0.891	0.855	0.654	0.636	0.682	0.563
INNE	0.730	0.860	0.847	0.645	0.638	0.669	0.551
CBLOF	0.726	0.815	0.823	0.608	0.606	0.650	0.511
SO-GAAL	0.721	0.797	0.818	0.569	0.667	0.544	0.489
AnoGAN	0.686	0.808	0.814	0.576	0.612	0.591	0.483
DIF	0.676	0.868	0.798	0.305	0.643	0.248	0.289
DeepSVDD	0.647	0.777	0.749	0.513	0.469	0.636	0.381
IForest	0.642	0.806	0.803	0.537	0.595	0.521	0.420
GMM	0.639	0.805	0.782	0.493	0.496	0.518	0.356
LODA	0.639	0.768	0.796	0.517	0.567	0.506	0.407
LUNAR	0.633	0.822	0.819	0.461	0.642	0.373	0.388
VAE	0.605	0.754	0.754	0.411	0.435	0.459	0.290
MO-GAAL	0.594	0.690	0.791	0.453	0.546	0.402	0.344
ECOD	0.577	0.775	0.796	0.478	0.594	0.446	0.388
COPOD	0.575	0.770	0.786	0.439	0.569	0.390	0.344
PCA	0.573	0.734	0.765	0.462	0.473	0.474	0.315
LMDD	0.479	0.650	0.812	0.315	1.000	0.202	0.384
ALAD	0.465	0.704	0.735	0.386	0.399	0.409	0.234
SOS	0.368	0.583	0.719	0.307	0.338	0.289	0.128

The results are averaged for every model, and sorted by *AUC*_*PR*_. The globally best results for each metric are **boldfaced**, and the best among the unsupervised algorithms are underlined.

## Usage Notes

The dataset^13^ contains the data in two different forms: a set of the original telemetry segments and a corresponding set of handcrafted features, both with anomaly labels. Both collections are also encoded in the popular, easy-to-handle CSV format and are ready to to use with various machine learning models (thus, they can be considered AI-ready). All the algorithms were implemented in Python using PyOD^23^ 1.1.2, TensorFlow^24^ 2.15, and PyTorch^25^ 2.1.2. Additionally, we used NumPy^26^ 1.26.2 and SciPy^27^ 1.13.1 and Pandas^28^ 2.1.4 for data preparation, Seaborn^29^ 0.13.0 for the visualizations, as well as OXI^16^ for the initial data analysis and labeling processes. Finally, our benchmark is accompanied with a Jupyter Notebook, containing an example experiment (modeling_examples.ipynb), in order to ensure the experimental reproducibility.

## Data Availability

The code for working with the OPS-SAT benchmark, including the functionalities used to prepare the numerical results, figures, and tables for this article, is available through the following GitHub repository: https://github.com/kplabs-pl/OPS-SAT-AD under the MIT license.

## References

[1] Pang, G., Shen, C., Cao, L. & Hengel, A. Deep learning for anomaly detection: A review. *ACM Computing Surveys (CSUR)* **54**, 1–38, 10.1145/3439950 (2021).

[2] Hundman, K., Constantinou, V., Laporte, C., Colwell, I. & Soderstrom, T. Detecting Spacecraft Anomalies Using LSTMs and Nonparametric Dynamic Thresholding. *Proceedings Of The 24th ACM SIGKDD International Conference On Knowledge Discovery & Data Mining*. pp. 387–395, 10.1145/3219819.3219845 (2018).

[3] Wu, R. & Keogh, E. Current Time Series Anomaly Detection Benchmarks are Flawed and are Creating the Illusion of Progress. *IEEE Transactions On Knowledge And Data Engineering* **35**, 2421–2429, 10.1109/TKDE.2021.3112126 (2023).

[4] Wagner, D. *et al*. TimeSeAD: Benchmarking Deep Multivariate Time-Series Anomaly Detection. *Transactions On Machine Learning Research*. https://openreview.net/forum?id=iMmsCI0JsS (2023).

[5] Amin Maleki Sadr, M., Zhu, Y. & Hu, P. An Anomaly Detection Method for Satellites Using Monte Carlo Dropout. *IEEE Transactions On Aerospace And Electronic Systems* **59**, 2044–2052, 10.1109/TAES.2022.3206257 (2023).

[6] Petković, M. *et al*. Machine-learning ready data on the thermal power consumption of the Mars Express Spacecraft. *Scientific Data* **9**, 229, 10.1038/s41597-022-01336-z (2022). 35610234 10.1038/s41597-022-01336-z PMC9130140

[7] Sanchez, F. *et al*. WebTCAD: A Tool for Ad-hoc Visualization and Analysis of Telemetry Data for Multiple Missions. *2018 SpaceOps Conference*. 10.2514/6.2018-2616 (2018).

[8] Kotowski, K., Haskamp, C., Ruszczak, B., Andrzejewski, J. & Nalepa, J. Annotating large satellite telemetry dataset for ESA international AI anomaly detection benchmark. *Proceedings Of The 2023 Conference On Big Data From Space (BiDS’23) - From Foresight To Impact - 6-9 November 2023, Austrian Center, Vienna, 2023*. pp. 341–344, 10.2760/46796 (2023).

[9] Kotowski, K. *et al*. European Space Agency Benchmark for Anomaly Detection in Satellite Telemetry. arXiv http://arxiv.org/abs/2406.17826 (2024).

[10] Evans, D. OPS-SAT: FDIR Design on a Mission that Expects Bugs - and Lots of Them. *SpaceOps 2016 Conference*. 10.2514/6.2016-2481 (2016).

[11] Ruszczak, B. *et al*. Machine Learning Detects Anomalies in OPS-SAT Telemetry. *Computational Science - ICCS 2023*. pp. 295–306, (2023).

[12] Kapoor, S. & Narayanan, A. Leakage and the reproducibility crisis in machine-learning-based science. *Patterns* **4**, 100804, 10.1016/j.patter.2023.100804 (2023). 37720327 10.1016/j.patter.2023.100804 PMC10499856

[13] Ruszczak, B., Kotowski, K., Nalepa, J. & Evans, D. OPSSAT-AD - anomaly detection dataset for satellite telemetry. *Zenodo*. 10.5281/zenodo.12588358 (2024).

[14] Shendy, R. & Nalepa, J. Few-shot satellite image classification for bringing deep learning on board OPS-SAT. *Expert Systems With Applications* **251**, 123984, 10.1016/j.eswa.2024.123984 (2024).

[15] ESA WebMUST - web client for OPS-SAT directory. (European Space Agency, 2021), https://opssat1.esoc.esa.int/webclient-must.

[16] Ruszczak, B., Kotowski, K., Andrzejewski, J., Haskamp, C. & Nalepa, J. OXI: An online tool for visualization and annotation of satellite time series data. *SoftwareX* **23**, 101476, 10.1016/j.softx.2023.101476 (2023).

[17] Nalepa, J. *et al*. Toward On-Board Detection Of Anomalous Events From OPS-SAT Telemetry Using Deep Learning. *8th International Workshop On On-Board Payload Data Compression*. 10.5281/zenodo.7244991 (2022).

[18] Nalepa, J. *et al*. Look ma, no ground truth! On building supervised anomaly detection from OPS-SAT telemetry. *Proceedings Of The International Astronautical Congress, 2023, International Astronautical Federation*. pp. 1–8, https://dl.iafastro.directory/event/IAC-2023/paper/78668/ (2023).

[19] Zhang, J. *et al*. Feature interpolation convolution for point cloud analysis. *Computers & Graphics* **99**, 182–191, 10.1016/j.cag.2021.06.015 (2021).

[20] Lubba, C. *et al*. catch22: CAnonical Time-series CHaracteristics. *Data Mining And Knowledge Discovery* **33**, 1821–1852, 10.1007/s10618-019-00647-x (2019).

[21] Tafazoli, S. *et al*. C22MP: the marriage of catch22 and the matrix profile creates a fast, efficient and interpretable anomaly detector. *Knowledge And Information Systems*. 10.1007/s10115-024-02107-5 (2024).

[22] Baldi, P., Brunak, S., Chauvin, Y., Andersen, C. & Nielsen, H. Assessing the accuracy of prediction algorithms for classification: an overview. *Bioinformatics* **16**, 412–424, 10.1093/bioinformatics/16.5.412 (2000). 10871264 10.1093/bioinformatics/16.5.412

[23] Zhao, Y., Nasrullah, Z. & Li, Z. PyOD: A Python Toolbox for Scalable Outlier Detection. *Journal Of Machine Learning Research* **20**, 1–7 (2019).

[24] Abadi, M. *et al*. TensorFlow: Large-Scale Machine Learning on Heterogeneous Systems. https://www.tensorflow.org/ (2015).

[25] Paszke, A. *et al*. Automatic differentiation in PyTorch (2017).

[26] Harris, C. *et al*. Array programming with NumPy. *Nature* **585**, 357–362, 10.1038/s41586-020-2649-2 (2020). 32939066 10.1038/s41586-020-2649-2 PMC7759461

[27] Virtanen, P. *et al*. Fundamental Algorithms for Scientific Computing in Python. *Nature Methods* **17**, 261–272, 10.1038/s41592-019-0686-2 (2020). 32015543 10.1038/s41592-019-0686-2 PMC7056644

[28] The Pandas development team pandas-dev/pandas: Pandas. Zenodo10.5281/zenodo.3509134 (2020).

[29] Waskom, M. Seaborn: statistical data visualization. *Journal Of Open Source Software* **6**, 3021, 10.21105/joss.03021 (2021).

[30] Grüning, M. & Kropf, S. A Ridge Classification Method for High-dimensional Observations. *From Data And Information Analysis To Knowledge Engineering*. pp. 684–691, 10.1007/3-540-31314-1_84 (2006).

[31] Fan, R., Chang, K., Hsieh, C., Wang, X. & Lin, C. LIBLINEAR: A Library for Large Linear Classification. *Journal Of Machine Learning Research* **9**, 1871–1874 (2008).

[32] Hastie, T., Rosset, S., Zhu, J. & Zou, H. Multi-class AdaBoost. *Statistics And Its Interface* **2**, 349–360, 10.4310/SII.2009.v2.n3.a8 (2009).

[33] Lee, C. & Lin, C. A study on l2-loss (squared hinge-loss) multiclass SVM. *Neural Comput.* **25**, 1302–1323, 10.1162/NECO_a_00434 (2013). 23470126 10.1162/NECO_a_00434

[34] Zhao, Y. & Hryniewicki, M. XGBOD: Improving Supervised Outlier Detection with Unsupervised Representation Learning. *2018 International Joint Conference On Neural Networks (IJCNN)*. pp. 1–8, 10.1109/IJCNN.2018.8489605 (2018).

[35] Kwon, D. *et al*. A survey of deep learning-based network anomaly detection. *Cluster Computing* **22**, 949–961, 10.1007/s10586-017-1117-8 (2019).

[36] Mastrangelo, C., Runger, G. & Montgomery, D. Statistical process monitoring with principal components. *Quality And Reliability Engineering International* **12**, 203–210 (1996).

[37] Aggarwal, C. Linear Models for Outlier Detection. *Outlier Analysis*. pp. 65–110, 10.1007/978-3-319-47578-3_3 (2017).

[38] Arning, A., Agrawal, R. & Raghavan, P. A Linear Method for Deviation Detection in Large Databases. *Knowledge Discovery And Data Mining* **1141**, 972–981 (1996).

[39] Tang, J., Chen, Z., Fu, A. & Cheung, D. Enhancing Effectiveness of Outlier Detections for Low Density Patterns. *Advances In Knowledge Discovery And Data Mining*. pp. 535–548, 10.1007/3-540-47887-6_53 (2002).

[40] Angiulli, F. & Pizzuti, C. Fast Outlier Detection in High Dimensional Spaces. *Principles Of Data Mining And Knowledge Discovery*. pp. 15–27, 10.1007/3-540-45681-3_2 (2002).

[41] He, Z., Xu, X. & Deng, S. Discovering cluster-based local outliers. *Pattern Recognition Letters* **24**, 1641–1650, 10.1016/S0167-8655(03)00003-5 (2003).

[42] Kriegel, H., Schubert, M. & Zimek, A. Angle-based outlier detection in high-dimensional data. *Proceedings Of The 14th ACM SIGKDD International Conference On Knowledge Discovery And Data Mining*. pp. 444–452, 10.1145/1401890.1401946 (2008).

[43] Liu, F., Ting, K. & Zhou, Z. Isolation Forest. *2008 Eighth IEEE International Conference On Data Mining*. pp. 413–422, 10.1109/ICDM.2008.17 (2008).

[44] Kriegel, H., Kröger, P., Schubert, E. & Zimek, A. Outlier Detection in Axis-Parallel Subspaces of High Dimensional Data. *Advances In Knowledge Discovery And Data Mining*. pp. 831–838, 10.1007/978-3-642-01307-2_86 (2009).

[45] Janssens, J., Huszár, F., Postma, E. & Herik, H. Stochastic outlier selection. (Tilburg University, Center for Cognition,2012)

[46] Kingma, D. & Welling, M. Auto-Encoding Variational Bayes. *2014 International Conference On Learning Representations (ICLR)*. **abs/1312.6114**, 10.48550/arXiv.1312.6114 (2013).

[47] Erfani, S., Rajasegarar, S., Karunasekera, S. & Leckie, C. High-dimensional and large-scale anomaly detection using a linear one-class SVM with deep learning. *Pattern Recognition*. **58** pp. 121–134, https://www.sciencedirect.com/science/article/pii/S0031320316300267 (2016).

[48] Pevný, T. Loda: Lightweight on-line detector of anomalies. *Machine Learning* **102**, 275–304, 10.1007/s10994-015-5521-0 (2016).

[49] Schlegl, T., Seeböck, P., Waldstein, S., Schmidt-Erfurth, U. & Langs, G. Unsupervised Anomaly Detection with Generative Adversarial Networks to Guide Marker Discovery. *Information Processing In Medical Imaging*. pp. 146–157 (2017)

[50] Ruff, L. *et al*. Deep One-Class Classification. *Proceedings Of The 35th International Conference On Machine Learning* **80**, 4393–4402 (2018).

[51] Zenati, H., Romain, M., Foo, C., Lecouat, B. & Chandrasekhar, V. Adversarially Learned Anomaly Detection. *2018 IEEE International Conference On Data Mining (ICDM)*. pp. 727–736, 10.1109/ICDM.2018.00088 (2018).

[52] Bandaragoda, T. *et al*. Isolation-based anomaly detection using nearest-neighbor ensembles. *Computational Intelligence* **34**, 968–998, 10.1111/coin.12156 (2018).

[53] Liu, Y. *et al*. Generative Adversarial Active Learning for Unsupervised Outlier Detection. *IEEE Transactions On Knowledge And Data Engineering* **32**, 1517–1528, 10.1109/TKDE.2019.2905606 (2020).

[54] Li, Z., Zhao, Y., Botta, N., Ionescu, C. & Hu, X. COPOD: Copula-Based Outlier Detection. *2020 IEEE International Conference On Data Mining (ICDM)*. pp. 1118–1123, (2020)

[55] Li, Z. *et al*. ECOD: Unsupervised Outlier Detection Using Empirical Cumulative Distribution Functions. *IEEE Trans. On Knowl. And Data Eng.* **35**, 12181–12193, 10.1109/TKDE.2022.3159580 (2022).

[56] Goodge, A., Hooi, B., Ng, S. & Ng, W. LUNAR: Unifying Local Outlier Detection Methods via Graph Neural Networks. *Proceedings Of The AAAI Conference On Artificial Intelligence* **36**, 6737–6745, 10.1609/aaai.v36i6.20629 (2022).

[57] Xu, H., Pang, G., Wang, Y. & Wang, Y. Deep Isolation Forest for Anomaly Detection. *IEEE Transactions On Knowledge And Data Engineering* **35**, 12591–12604, 10.1109/TKDE.2023.3270293 (2023).

