# Correction: The OPS-SAT benchmark for detecting anomalies in satellite telemetry

**DOI:** 10.1038/s41597-025-05751-w

**Published:** 2025-08-18

**Authors:** Bogdan Ruszczak, Krzysztof Kotowski, David Evans, Jakub Nalepa

**Affiliations:** 1 Computer Science Department, Opole University of Technology, Prószkowska Str. 76, 45-758 Opole, Poland; 2 KP Labs, Bojkowska Str. 37J, 44-100 Gliwice, Poland; 3 European Space Agency/ESOC, Robert-Bosch-Str. 5, 64293 Darmstadt, Germany; 4 Faculty of Automatic Control, Electronics and Computer Science, Department of Algorithmics and Software, Silesian University of Technology, Akademicka Str. 16, 44-100 Gliwice, Poland

**Keywords:** Computational science, Information technology

Correction to: *Scientific Data* 10.1038/s41597-025-05035-3, published online 29 April 2025

In this article several of the figures were inadvertently interchanged. While the figure numbers and labels are accurate, Figures 3, 4, 5, 6, 7, 8, 9, and 10 are not in the correct order. The original article has been corrected.

